# Expression of the peanut diacylglycerol acyltransferase 3 increases the neutral lipid content and improves the fatty acid composition of *Chlorella vulgaris*

**DOI:** 10.3389/fpls.2026.1750015

**Published:** 2026-03-05

**Authors:** Moran Topf, Anna Andreeva, Helen Saul, Tamar Tenenvorzel, Lotem Davidi-Shwarts, Zehavit Dadon, Irina Berezin, Yael Kinel-Tahan, Yaron Yehoshua, Orit Shaul

**Affiliations:** The Mina and Everard Goodman Faculty of Life Sciences, Bar-Ilan University, Ramat-Gan, Israel

**Keywords:** biofuel, *Chlorella vulgaris*, diacylglycerol acyltransferase 3 (DGAT3), fatty acid, lipid, microalgae, triacylglycerol (TAG)

## Abstract

Microalgae are a potential source of renewable biofuel with several advantages over conventional crops. Under stress conditions, oleaginous microalgae such as *Chlorella vulgaris* accumulate high levels of neutral lipids, mainly in the form of triacylglycerol (TAG), which can be converted into biodiesel. However, the growth under stress conditions limits biomass accumulation. DGAT enzymes catalyze the final step in TAG biosynthesis, by transferring a fatty acyl-CoA to diacylglycerol. We describe here the first case in which a higher plants DGAT3-type enzyme has been overexpressed in an oleaginous microalga. Higher plants DGAT3 enzymes differ in their properties from other types of DGAT enzymes and also from the distantly related group of enzymes nominated DGAT3 in algae. We overexpressed in *C. vulgaris* the DGAT3 of *Arachis hypogaea* (peanut), since this enzyme utilizes oleoyl-CoA as the preferred acyl donor. Oleic acid is a favorable fatty acid constituent of biofuel due to its low melting point and a relatively low vulnerability to oxidation. The sequence and regulatory regions of *AhDGAT3* were optimized for supporting efficient expression. The transformed algal lines showed up to a five-fold increase in the content of neutral lipids. This increase occurred under normal growth conditions, which do not limit biomass accumulation. The transformed algae also showed a four-fold increase in the percentage of oleic acid and a 25% reduction in the percentage of linolenic acid among the lipid-derived fatty acids. Both changes are favorable for biodiesel utilization. This work demonstrates that higher plants DGAT3 enzymes, and particularly the peanut DGAT3, can be utilized for obtaining improved microalgal feedstocks for biofuel production.

## 1 Introduction

The depletion of fossil fuel reserves and the harmful environmental effects associated with their utilization are a global concern. This necessitates the development of renewable energy sources, including biofuels. Microalgae are a potential renewable biofuel source with several advantages over conventional crops (Katiyar et al., 2017). Microalgae annual oil production per hectare of land could be up to 100-fold greater than that of higher plants (Hu et al., 2008). Microalgae do not compete for land, fresh water or fertilizer with agricultural food production, thus avoiding the increases in food prices due to utilization of oil crops. Algal growth increases global CO_2_ fixation. Biofuel produced from microalgae has benefits of 78% decline in CO_2_ emission, 98% decline in sulfur emissions and a 50% decline in particulate matter after combustion compared to petroleum diesel (Banerjee et al., 2016).

Several studies have established that microalgae cultivation for biodiesel production as a single objective will not be economically feasible due to the cost of growth, nutrients and harvest. However, later studies showed that microalgal growth for simultaneous biodiesel production and a second utilization, such as industrial CO_2_ mitigation or wastewater remediation, could be economically feasible (Jayakumar et al., 2017; Chen et al., 2018; Fazal et al., 2018). The factor that has the greatest effect on the biodiesel production cost is algal oil content, followed by the factor of annual productivity (which depends on biomass accumulation) (Brownbridge et al., 2014). Thus, it is desired to increase algal oil content while using growth regimes that do not limit biomass accumulation.

Under stress conditions, oleaginous algae accumulate high levels of neutral lipids, mainly in the form of triacylglycerol (TAG), which can be converted into biodiesel (Hu et al., 2008). However, these algae accumulate high levels of neutral lipids only under stress condition, such as nitrogen starvation. Consequently, lipid production from natural oleaginous microalgae is carried out in a two-phase process, in which a growth phase under normal conditions is followed by stress conditions, typically nitrogen starvation. However, these stress conditions inhibit biomass accumulation (e.g., Gao et al., 2023), reducing the potential productivity (Brownbridge et al., 2014). To reduce the cost of algal biofuel, it is essential to obtain microalgae producing increased neutral lipid levels under normal growth conditions, without nitrogen starvation or another stress.

Important properties that affect the suitability of any material as diesel fuel are viscosity, cold flow (the behavior at low temperatures), and oxidative stability (Knothe, 2009). These properties are largely determined by the fatty acid (FA) composition of the TAG in the biodiesel feedstock (Knothe, 2009). Most common biodiesel feedstocks (e.g., vegetable oils and animal fats) consist mainly of the following FAs: palmitic acid (16:0), stearic acid (18:0), oleic acid (18:1), linoleic acid (18:2) and linolenic acid (18:3). Biodiesel derived from these feedstocks will face the problems of poor cold flow properties or insufficient oxidative stability or, in most cases, both (Knothe, 2009). A higher melting point means lower ability to use the biodiesel at low temperatures. Both the viscosity and the melting point of a biodiesel increase with higher chain length and higher saturation of the FAs of the feedstock. Double bonds in the FA chains can undergo oxidation. Relative rates of oxidation are 1 for oleate (18:1), 41 for linoleate (18:2), and 98 for linolenate (18:3). Thus, polyunsaturated FAs are not favorable as biodiesel constituent. Due to the balance between the viscosity, cold flow, and oxidative stability demands, the preferred FAs in TAG of biodiesel feedstocks are palmitoleic acid (16:1) and oleic acid (18:1) (Knothe, 2009).

The pathway of TAG biosynthesis in microalgae, which is generally similar to that of higher plants, is described by Chen and Smith, 2012; Klok et al., 2014; Xu et al., 2018; and Pavlenko et al., 2022. FAs are synthesized *de novo* in plastids, where they are utilized for producing diacylglycerol (DAG) and plastid membrane lipids. Some of the FAs are exported to the endoplasmic reticulum (ER) and utilized for DAG production there. The enzyme diacylglycerol acyltransferase (DGAT) catalyzes the formation of TAG from DAG and a fatty acyl-CoA. This reaction is the final and committed step in *de novo* TAG biosynthesis (Cao, 2011; Cai et al., 2025). An increase in DGAT expression is one of the main changes in gene expression occurring in oleaginous microalgae during the switch into rapid TAG synthesis under nitrogen starvation (Guarnieri et al., 2011; Boyle et al., 2012; Li et al., 2014; Fan et al., 2014; Jia et al., 2015; Arora et al., 2018; Gao et al., 2023). It was thus rationalized that DGAT overexpression could increase TAG production in microalgae.

The main DGAT types identified in higher plants include type-1 and type-2 DGATs (referred to hereafter as DGAT1 and DGAT2), which are ER-localized integral membrane proteins, and type-3 DGAT enzymes (DGAT3) that are soluble cytosolic enzymes (Saha et al., 2006; Lung and Weselake, 2006; Cao, 2011; Hernandez et al., 2012; Liu et al., 2012; Rani et al., 2013). DGATs of different types have different biochemical properties and substrate preferences (Shockey et al., 2006; Cao, 2011; Liu et al., 2012; Turchetto-Zolet et al., 2016; Xu et al., 2018; Pavlenko et al., 2022). Previous attempts to increase lipid levels in microalgae by genetic engineering had mixed results (reviewed by Klok et al., 2014; Banerjee et al., 2016; Remmers et al., 2018; and Xu et al., 2018). Most attempts to overexpress DGAT enzymes in microalgae were related to DGAT2 (see Discussion). The changes observed in the FA profile of the transformed algae reflected the fact that DGAT2 enzymes have substrate preferences for polyunsaturated FAs (Liu et al., 2012), which are not favorable as biodiesel constituent (Knothe, 2009). A few studies also described the overexpression of DGAT1 enzymes in microalgae (see Discussion).

Higher plants DGAT3 were identified and functionally characterized in several plant species (Saha et al., 2006; Hernandez et al., 2012; Cao et al., 2013; Chi et al., 2014; Turchetto-Zolet et al., 2016; Ayme et al., 2018; Zhao et al., 2021; Gao et al., 2021; Han et al., 2022; Xue et al., 2022; Zhou et al., 2025). Several studies described the expression of higher plants DGAT3 enzymes in higher plants. Expression of peanut (*Arachis hypogaea*) DGAT3 in *Arabidopsis thaliana* and soybean (*Glycine max*) increased the total FA content of their seeds by 10 and 4%, respectively (Xu et al., 2023). The content of oleic acid (18:1) in the seeds was increased by 35 and 25%, respectively. Expression of cotton (*Gossypium hirsutum*) DGAT3 in *A. thaliana* increased three fold the seed oil content, with a 10% increase in 18:1 levels (Zhao et al., 2021). Expression in tobacco (*Nicotiana benthamiana*) of *Camelina sativa* DGAT3 increased by 28% the seed oil content (Gao et al., 2021). Overexpression in *A. thaliana* of *Paeonia rockii* DGAT3 increased by 50% the total leaf FA content, with a reduction in 18:1 content and an increase in the content of 18:2 and 18:3 (Han et al., 2022). Expression of soybean DGAT3 in tobacco increased by 17% the oil content of seeds, with a 70% increase in the 18:1 content of the seed TAGs (Xue et al., 2022). In a recent report, the DGAT3 of the higher plant *Perilla frutescens* was expressed in the non-oleaginous alga *Chlamydomonas reinhardtii*. However, this enzyme, which has preference to the 18:3 FA, increased the content of 18:3 in *C. reinhardtii* by 21%, and reduced the levels of the preferable FAs 18:1 and 16:1 as compared to the untransformed algae (Zhou et al., 2025).

Higher plants DGAT3 enzymes had not been previously overexpressed in an oleaginous microalga. Here we investigated the ability of the peanut DGAT3 (AhDGAT3) enzyme to affect the lipid content and the FA composition of an oleaginous microalga, *Chlorella vulgaris*, under normal growth conditions. *C. vulgaris* is of industrial interest due to its high growth rate and its potential to reach a high lipid content when grown under stress conditions such as nitrogen starvation (Wang et al., 2012; Anthony et al., 2015). The peanut DGAT3 was selected for this study since for this enzyme, oleoyl-CoA is the preferred acyl donor (Saha et al., 2006). As indicated above, oleic acid (18:1) is a favorable biodiesel constituent due to its low melting point and a relatively low vulnerability to oxidation (Knothe, 2008). While it was possible that the FA composition of the host alga would determine the availability of FAs for TAG production, we postulated that elevated utilization of oleoyl-CoA by the introduced AhDGAT3 enzyme might increase the production of this FA. The results demonstrate that AhDGAT3 overexpression in the oleaginous microalga *C. vulgaris* significantly increased the neutral lipid content of algae grown under normal conditions. Moreover, this was accompanied by a significant increase in the percentage of oleic acid and a reduction in the percentage of linolenic acid among the lipid-derived FAs. Both changes are favorable for biodiesel production. This shows the potential of utilizing AhDGAT3 for obtaining valuable microalgal feedstocks for biofuel production.

## 2 Materials and methods

### 2.1 Generation of constructs and algal transformation

**Figure 1:**
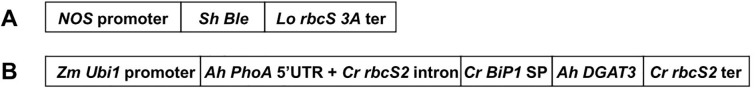
The constructs used for *C. vulgaris* transformation. **(A)** The chimeric selectable marker gene. *NOS* promoter - the promoter of the *nopaline synthase* gene of *A. tumefaciens*, *Sh ble* - the coding sequence of the bleomycin resistance protein of *S. hindustanus*, *Lo rbcS 3A* ter - the terminator of pea (*L. oleraceus*) *rbcS-3A* gene. **(B)** The chimeric gene for DGAT3 expression. *Zm Ubi1* promoter - the promoter of maize (*Z. mays*) *ubiquitin 1* gene, *At PhoA* 5’UTR + *Cr rbcS* intron - the 5’ UTR of the *A. thaliana PhoA* gene, in which the first intron of the *C. reinhardtii rbcS2* gene was inserted, *Cr BiP1* SP - the signal peptide of the *C. reinhardtii BiP1* gene, *Ah DGAT3* - the coding sequence of peanut (*A. hypogaea*) *DGAT3* gene, to which we added the coding sequence of the HDEL amino acids (which is an ER retention signal) at the C terminus, *Cr rbcS*2 ter - the terminator of the *C. reinhardtii rbcS2* gene.

The constructs created for expression in *C. vulgaris* are presented in **Figure 1**. All sequences included in these constructs were synthesized by Biomatik, and are presented in **Supplementary Figure S1**.

The chimeric gene created for expression of *Streptoalloteichus hindustanus ble* in *C. vulgaris* is presented in **Figure 1A**. The enzyme encoded by this gene confers zeocin resistance. The *Sh ble* coding sequence was cloned under the control of the *Agrobacterium tumefaciens nopaline synthase* (*NOS*) promoter, which was shown to mediate efficient expression in *C. reinhardtii* (Diaz-Santos et al., 2013), and the pea (*Lathyrus oleraceus*) *rbcS-3A* gene terminator.

The chimeric gene created for expression of *AhDGAT3* in *C. vulgaris* is presented in **Figure 1B**. Since algae often have an unusual codon usage (Tabatabaei et al., 2011), the coding sequence of *AhDGAT3* was optimized according to the *C. vulgaris* codon usage. *AhDGAT3* coding sequence was expressed under the control of the maize (*Zea mays*) ubiquitin (*Ubi1*) promoter, which was shown to mediate high expression in *Chlorella* (Chen et al., 2001), and the *C. reinhardtii rbcS2* gene terminator. We also used the 5’ untranslated region (5’ UTR) of the *A. thaliana PhoA* gene, which was shown to be a very efficient enhancer of translation (Agarwal et al., 2014). Introns, particularly those localized in the 5’ UTR, can significantly increase gene expression (reviewed by Shaul, 2017). We thus inserted in the *PhoA* 5’ UTR the first intron of the *C. reinhardtii rbcS* gene, which was shown to increase transgene expression in *C. reinhardtii* (Eichler-Stahlberg et al., 2009). To further promote efficient expression, the flanking sequences of *AhDGAT3* were optimized according to the Kozak consensus for efficient translation and for efficient recognition of the transcription initiation site. AhDGAT3 is a soluble enzyme (Saha et al., 2006). To target AhDGAT3 to the ER, we fused the signal peptide (SP) of the *C. reinhardtii BiP1* gene at its N terminus, and an ER retention signal (HDEL) at its C terminus. These signal elements were previously shown to successfully target foreign soluble proteins into the *C. reinhardtii* ER (Rasala et al., 2014).

The two constructs presented in **Figure 1** were cloned into an *A. tumefaciens* binary vector, immobilized into *A. tumefaciens* strain EHA105, and used for *C. vulgaris* transformation as previously described (Cha et al., 2012; Sharif et al., 2015). Transformed algae were selected on Bristol x2 (Bold, 1949) agar plates including 6 µg ml^-1^ zeocin.

### 2.2 Growth conditions

*C. vulgaris* (strain number 211-11b from the SAG culture collection, University of Göttingen, Germany) was grown in a climate-controlled growth room at 22°C with a photoperiod of 12 h light and 12 h darkness. Each algal line was separately propagated and maintained on agar plates including Bristol x2 medium (Bold, 1949), which included 6 µg ml^-1^ zeocin for the transformed lines. The lines were also maintained in liquid TAP medium (Gorman and Levine, 1965) supplemented with 1 gr L^-1^ glucose.

Algae grown in photoautotrophic conditions were maintained in liquid Bristol x1 medium with continuous bubbling of filtered fresh air. Algae grown in mixotrophic conditions were maintained in liquid TAP medium supplemented with 1 gr L^-1^ glucose, in closed vessels, with continuous shaking. The growth conditions during the experiments for determination of lipid content and FA composition were as follows. For photoautotrophic growth conditions, stationary phase algae were diluted in a fresh medium to an initial OD 750 nm of 0.25, and maintained in 2 L flasks including 600 ml medium containing 500 mg/ml cefotaxime for 19 days. For mixotrophic growth conditions, stationary phase algae were diluted in a fresh medium to an initial OD 750 nm of 0.05, and maintained in 5 L flasks including 1 L medium containing 500 mg/ml cefotaxime for five days.

Comparison of growth under nitrogen replete and deplete conditions was carried out as follows. Stationary phase algae were diluted and grown in Bristol x2 medium, which included nitrogen, in 250 ml flasks including 50 ml medium containing 500 mg/ml cefotaxime, with constant aeration. After four days the algae were centrifuged and resuspended into equal densities (as determined by OD 750 nm measurements) in Bristol x1 medium containing 500 mg/ml cefotaxime that either included (+N) or did not include (-N) nitrogen, and were grown for additional five days.

### 2.3 Determination of the duplication times of the algal cultures

Algae were grown in mixotrophic conditions in 50 ml medium in 250 ml flasks with constant shaking. Stationary-phase algae were diluted to an initial OD 750 nm of 0.006. Samples were collected every 8-16 h and their OD 750 nm was determined. We found that the OD 750 nm of the cultures was proportional to their cell number, as determined by a hemocytometer. The duplication time (DT) of each culture (average number of hours required for cell division) was determined during the logarithmic growth phase by the following equation: DT=h/[ln(OD_f_/OD_i_)/ln(2)], where h is the time interval in hours between two measurements, and OD_f_ and OD_i_ are the OD 750 nm measurements at the final and initial time points, respectively.

### 2.4 RNA extraction and reverse transcriptase PCR

Total RNA was extracted from algal samples with the TRI-Reagent (Merck) according to the manufacturer’s instructions. RNA was treated with DNase I, followed by DNase I removal, using Ambion’s AM1906 kit according to the manufacturer’s instructions. A preliminary PCR reaction with the same primers that were subsequently used for RT-PCR was conducted to verify that no DNA remained. Preparation of cDNA was carried out using an oligo dT primer and M-MuLV reverse transcriptase (RevertAid™, Fermentas) according to the manufacturer’s instructions. This cDNA was utilized as a template for non-quantitative RT-PCR analyses of *AhDGAT3* and the *C. vulgaris* 18S rRNA sequences. For *AhDGAT3*, the primers used were: forward primer 5’- CGTGACATGCCCCTCCTTTA -3’ and reverse primer 5’- CTTCTTCTCGTCGCCGTAGT -3’, and the PCR conditions were as follows: an initial cycle of 94°C for 3 min, followed by 36 cycles of 94°C for 30 sec, 58°C for 30 sec, and 72°C for 30 sec, followed by a final cycle of 72°C for 10 min. For the 18S rRNA, the primers used were: forward primer 5’- CAGCCTGCTAAATAGTCACGG -3’ and reverse primer 5’- CAGAACATCTAAGGGCATCACA -3’, and the PCR conditions were as follows: an initial cycle of 95°C for 3 min, followed by 4 cycles of 95°C for 30 sec, 48°C for 30 sec, and 72°C for 30 sec, followed by 25 cycles of 95°C for 30 sec, 56°C for 30 sec, and 72°C for 30 sec, followed by a final cycle of 72°C for 10 min.

### 2.5 Quantitative determination of neutral lipid content

The content of neutral lipid was determined by an improved Nile red (NR) fluorescence method, whose results were previously shown to be identical to those obtained by a gravimetric method in *C. vulgaris* (Chen et al., 2009). The algal samples (125 µl) were placed in a black 96-well plate, and each well was supplemented with 125 µl of 25% DMSO including NR (the final concentration of NR was 12 µg ml^-1^). The plate was incubated in darkness for 1 hour at 40°C. The fluorescence was determined in a BioTek Synergy 4 Microplate Reader (Agilent) with the excitation wavelength set at 530 nm and the emission wavelength at 570 nm. If necessary, the algal samples were first diluted to a cell concentration range in which the NR fluorescence results were linearly correlated with cell concentration.

### 2.6 Determination of the fatty acid composition of algal lipids

Algal cells were grown as described in section 2.2, harvested by centrifugation, lyophilized, and lipids were extracted according to Folch et al. (1957). Ten ml of chloroform: methanol, 2:1 (v:v) were added to each dried algal sample. The mixture was incubated for 30 min at 37 °C with occasional mixing. After centrifuging for 10 min at 500xg, the supernatant was eliminated. The lower organic phase was filtered through Whatman #1 filter paper and water (20% of the volume) were added. After centrifuging for 10 min at 500xg, the lower chloroform phase was evaporated and utilized for fatty acid methyl esters (FAMEs) preparation according to Leikin-Frenkel et al. (2021). One ml boron trifluoride-methanol solution (14% in methanol, Sigma-Aldrich) was added to the dried samples for FA methylation. The tubes were gassed with nitrogen, closed tightly, and heated at 85°C for 45 min with occasional shaking. After cooling, 1 ml of hexane was added and after decantation, the hexane layer was transferred to new tubes. The hexane extracts were concentrated by evaporation under nitrogen. An aliquot (1 μl) of the final hexane resuspension was injected into a Varian 3800 Series gas chromatograph equipped with a flame ionization detector (FID). The FAMEs were separated using a fused-silica capillary column (SGE, 30 m x 0.25 mm i.d.). Data acquisition and processing were performed using the Varian Star Workstation (Advanced Application Software, version 6.x; Varian, Inc., Walnut Creek, CA). The FA retention times were compared for identification to those of the components of known commercial mixture of FAs, PUFA2 and PUTA3 (Supelco, USA). The level of each FA was calculated in µg by comparing its area to that of the internal standard 17:0 included in the same chromatogram, according to the formula: µg FA = (Area FA x µg 17:0)/Area 17:0. The FAs were expressed as µg percent of each individual FA in the total profile.

## 3 Results

### 3.1 Expression of AhDGAT3 in *C. vulgaris*

To identify a suitable antibiotic for selection of transformed *C. vulgaris* cells, algal growth was examined in the presence of several antibiotics. Both zeocin and hygromycin were suitable for selection of transformed algal cells (**Supplementary Figure S2A**). The optimal zeocin concentration for this selection was found to be 6 µg ml^-1^ (**Supplementary Figure S2B**). Zeocin resistance is conferred by the *S. hindustanus ble* gene. We thus created a chimeric gene for *Sh ble* expression in *C. vulgaris* (**Figure 1A**).

The chimeric gene created for expression of AhDGAT3 in *C. vulgaris* is presented in **Figure 1B**. Several approaches were used for optimization of AhDGAT3 expression, including codon usage optimization and selection of regulatory and consensus sequences that were previously shown to mediate efficient transcription and translation (see details in Materials and Methods). For targeting the soluble DGAT3 enzyme to the ER, the main site of TAG production, we fused to it targeting sequences that were previously shown to successfully target foreign soluble proteins into the *C. reinhardtii* ER (Rasala et al., 2014) (see details in Materials and Methods).

**Figure 2:**
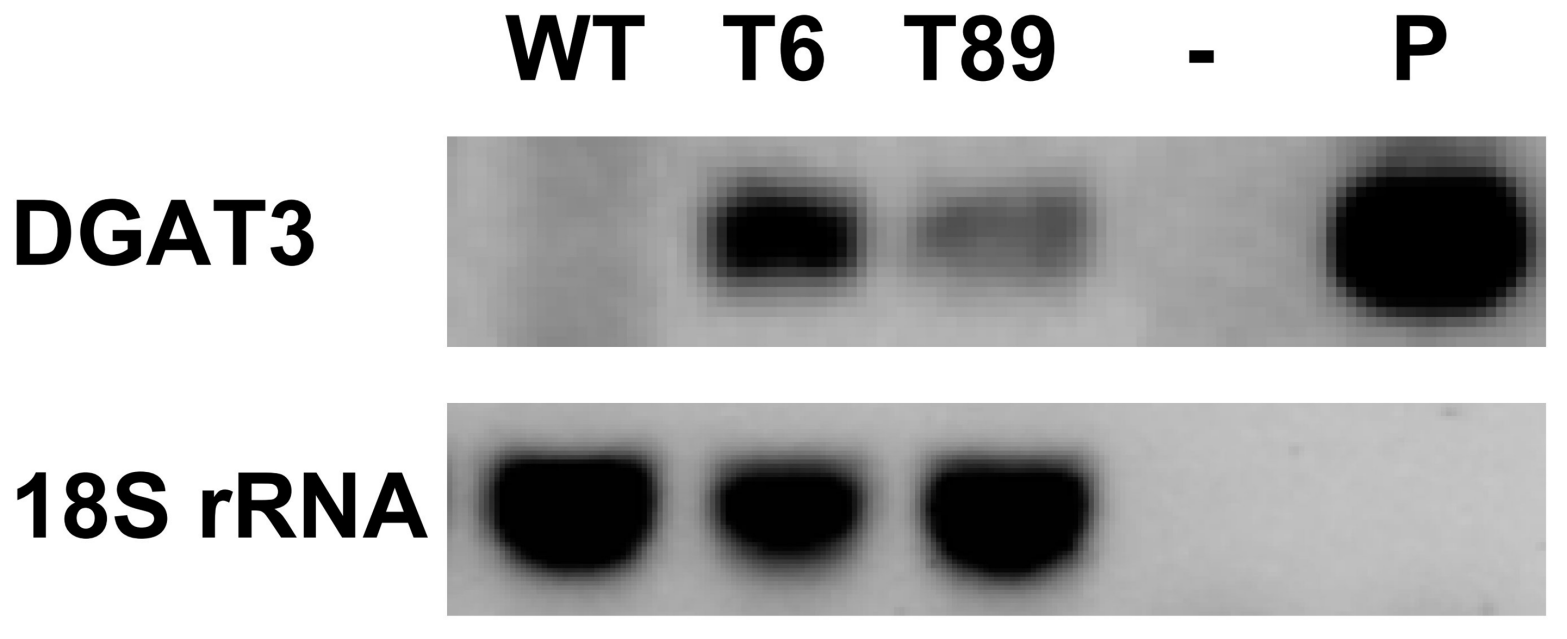
Non-quantitative reverse transcriptase PCR analysis of algae expressing the chimeric *AhDGAT3* gene. The cDNA prepared from the WT and transformed algae was utilized as a template for non-quantitative RT-PCR analysis of *AhDGAT3* and the *C. vulgaris* 18S rRNA sequences. The later sequence was utilized as a control for cDNA integrity. WT - untransformed *C. vulgaris*, T6 and T89 - two independent transformed *C. vulgaris* lines, the “-” indicates a negative control containing no template, P – a positive control with a plasmid including the chimeric *AhDGAT3* gene as a template.

The chimeric *DGAT3* and *ble* genes presented in **Figure 1** were transformed to *C. vulgaris* by *Agrobacterium*, and several zeocin resistant colonies were selected. Independent transformed lines were grown separately in liquid medium, and lines with increased levels of neutral lipids were screened by Nile red fluorescence. Two lines (T6 and T89) were selected for further analysis. The selected lines maintained their zeocin resistance throughout all this work (**Supplementary Figures 2C, D**). The expression of *AhDGAT3* in the selected lines was tested by non-quantitative reverse-transcriptase (RT) PCR. Both transformed lines showed a band with a size similar to that of a plasmid-derived *AhDGAT3* sequence, which was not observed in untransformed algae (**Figure 2**). This indicated that the chimeric *AhDGAT3* gene was expressed in the two selected transformed lines.

### 3.2 The transformed lines showed up to a five fold increase in their neutral lipid content

**Figure 3:**
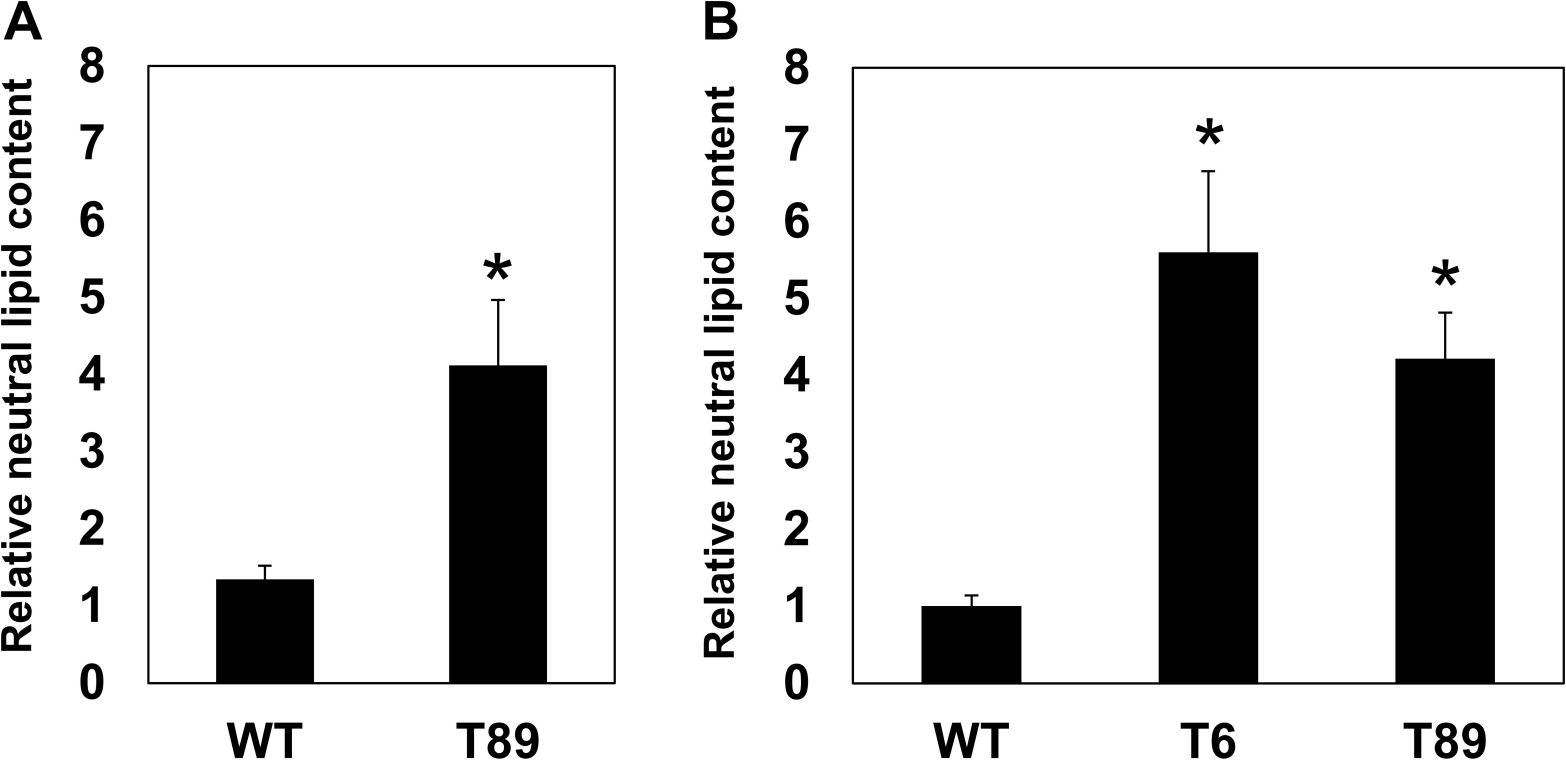
Relative content of neutral lipids in the transformed algae. **(A)** Algae were grown in photoautotrophic conditions for 19 days. **(B)** Algae were grown in mixotrophic conditions for five days. The content of neutral lipids was determined as described in the Materials and Methods. For both the photoautotrophic and mixotrophic conditions, the data were first normalized to the OD 750 nm of each culture (which is proportional to algal biomass) and then to the neutral lipid content of WT algae grown in the mixotrophic conditions (to allow the comparison between the two conditions). The graphs present the averages and standard errors of four independent experiments; each experiment included three independent flasks for each algal line in each condition. An asterisk indicates a statistically significant difference (*p* < 0.05) between the WT and the transformed lines, as determined by two-sided Student’s *t*-test. WT - untransformed algae, T6 and T89 - two independently transformed algal lines.

The transformed lines were subsequently grown in a series of experiments, and their neutral lipid content was analyzed by the improved Nile red (NR) fluorescence method of Chen et al. (2009). The results of this improved NR method were previously shown to be identical to those obtained by a gravimetric method in *C. vulgaris* (Chen et al., 2009). A high correlation (R^2^ above 0.97) between the results of the NR and the gravimetric methods was also demonstrated for *C. vulgaris* grown under different culture conditions (Huang et al., 2009) and for *Chlorella saccharophila* (Isleten-Hosoglu et al., 2012). The first experiments with the transformed algal lines were carried out in photoautotrophic conditions, in which the algae were not supplemented with organic carbon and their growth was absolutely dependent on photosynthesis. However, we realized that under these growth conditions, the rates of biomass and lipid accumulation were delayed compared to mixotrophic growth conditions (see below). In addition, the continuous bubbling of the photoautotrophic cultures (for aeration) for long periods made it difficult to avoid bacterial and fungal contamination. Thus, although increased lipid content was also obtained under photoautotrophic conditions (**Figure 2A** and **Supplementary Figure S3**), most analyses were carried out under mixotrophic conditions (**Figure 3B**). Under mixotrophic conditions, the cultures were grown in closed vessels under light, and thus could carry out photosynthesis, but were also supplemented with a small amount of organic carbon. In a series of four independent experiments, the transformed lines showed a 4 to 5.5 fold increase in the content of neutral lipids compared to untransformed algae (**Figure 3**).

### 3.3 The utilization of the transformed algae is advantageous over growing untransformed algae under nitrogen starvation

**Figure 4:**
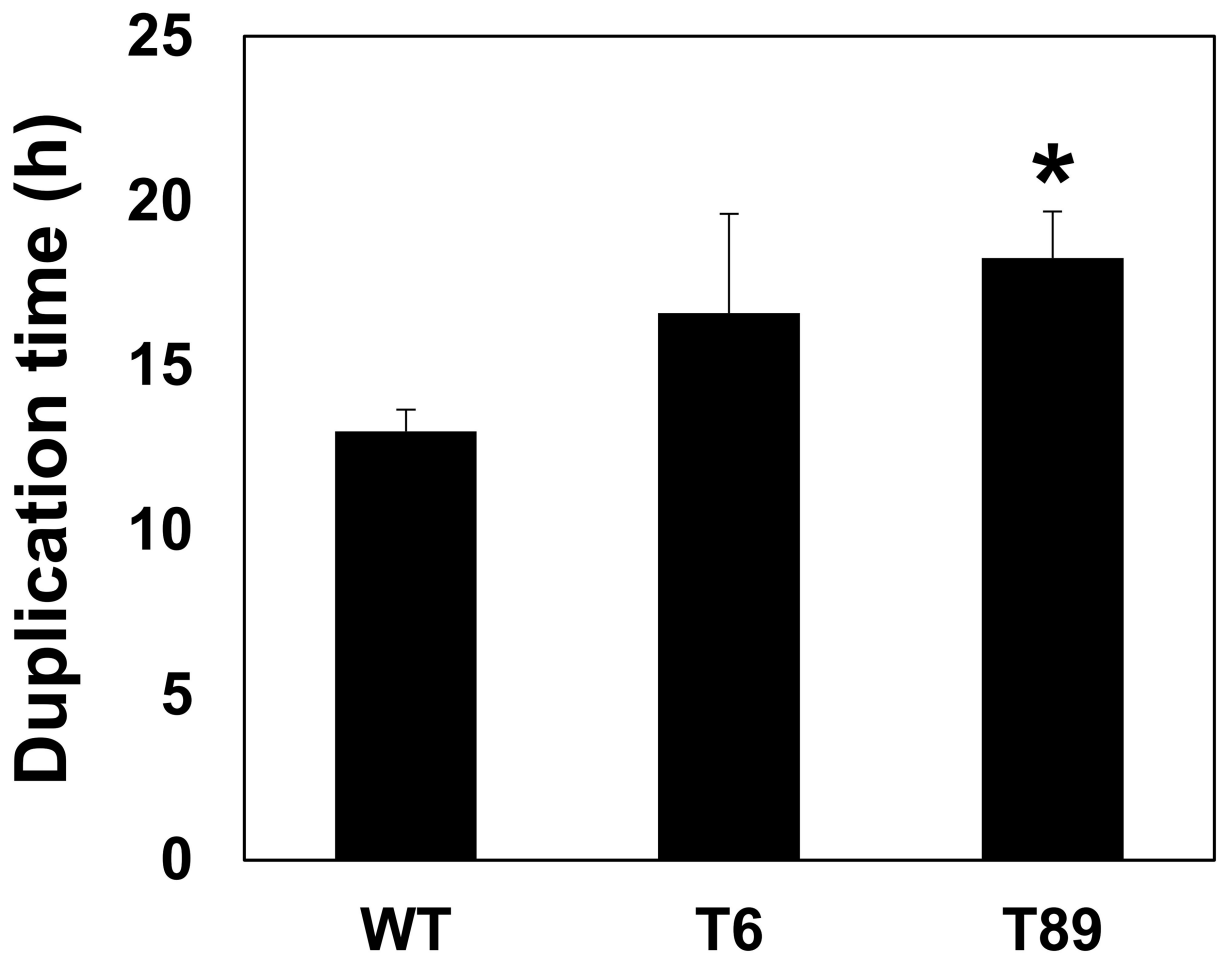
Duplication times of the algae grown in mixotrophic conditions. Each column presents the average and standard error of the duplication times that were determined and calculated as described in section 2.3 of the Meterials and Methods. The data presented were based on measurements at 3-4 time points of three independent flasks for each algal line. An asterisk indicates a statistically significant difference (*p* < 0.05) between the WT and the transformed lines, as determined by two-sided Student’s *t*-test. WT - untransformed algae, T6 and T89 - two independently transformed algal lines.

To assess the rate of biomass accumulation of the different lines, we determined the duplication times of the wild type (WT) and transformed algae. As shown in **Figure 4**, there was some increase in the duplication time of the transformed lines as compared to the WT algae, although the difference was statistically significant only for the T89 line.

**Figure 5:**
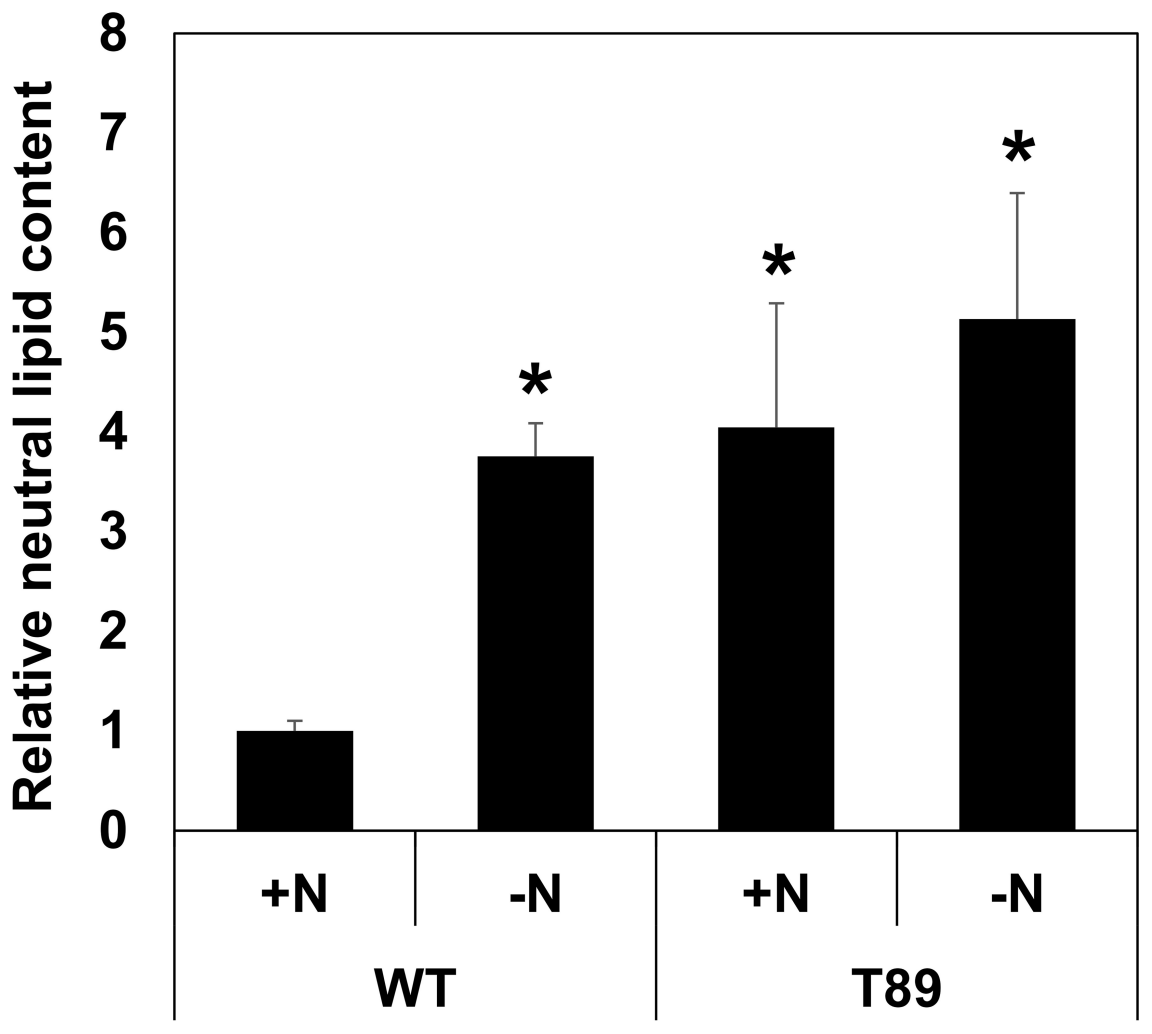
Relative content of neutral lipids for algae grown in nitrogen replete (+N) or deplete (-N) conditions (see Materials and Methods). The content of neutral lipids was determined as described in the Materials and Methods. The data were first normalized to the OD 750 nm of each culture (which is proportional to algal biomass) and then to the neutral lipid content of WT algae grown in +N conditions. The graph presents the averages and standard errors of two independent experiments; each experiment included three independent flasks for each algal line. An asterisk indicates a statistically significant difference (*p* < 0.05) between WT algae grown in +N conditions and the other growth conditions or algal lines, as determined by two-sided Student’s *t*-test. WT - untransformed algae, T89 - a transformed algal line.

As mentioned, the process of neutral lipid production from natural oleaginous microalgae is currently carried out in a two-phase process, in which a growth phase under normal conditions is followed by nitrogen starvation. However, nitrogen starvation inhibits biomass accumulation. It was thus interesting to compare the neutral lipid produced by WT and transformed algae in growth processes that involve or do not involve nitrogen starvation. For this, the algae were first grown in a nitrogen containing medium, then centrifuged and transferred to a nitrogen containing or nitrogen depleted medium, and then grown for additional few days to allow biomass and lipid accumulation (**Figure 5**). The process was carried out under photoautotrophic conditions, to avoid the potential manifestation of another stress condition due to possible depletion during the growth period of the organic carbon present in the mixotrophic medium. Similar to previous observations for oleaginous algae (Hu et al., 2008), the neutral lipid content of WT algae was significantly increased under nitrogen starvation (**Figure 5**). The increase factor observed for WT algae, 3.8 fold, was comparable to other observations in *C. vulgaris* and *Chlorella* sp., which demonstrated a 4-5 fold increase in the amount of lipids that could be obtained from a given culture biomass under nitrogen deplete *vs*. nitrogen replete conditions (Breuer et al., 2012; Topf et al., 2014). The neutral lipid contents of AhDGAT3 overexpressing algae grown under either nitrogen deplete or replete conditions were similar to that of WT algae grown under nitrogen starvation (**Figure 5**). This shows that, taking into consideration the reduction in culture biomass due to nitrogen starvation, the utilization of transformed algae and growth under nitrogen replete conditions is advantageous over the utilization of either the WT or transformed algae in growth processes that involve nitrogen starvation.

### 3.4 The transformed algal lipids have a favorable FA composition with increased oleic acid levels

**Figure 6:**
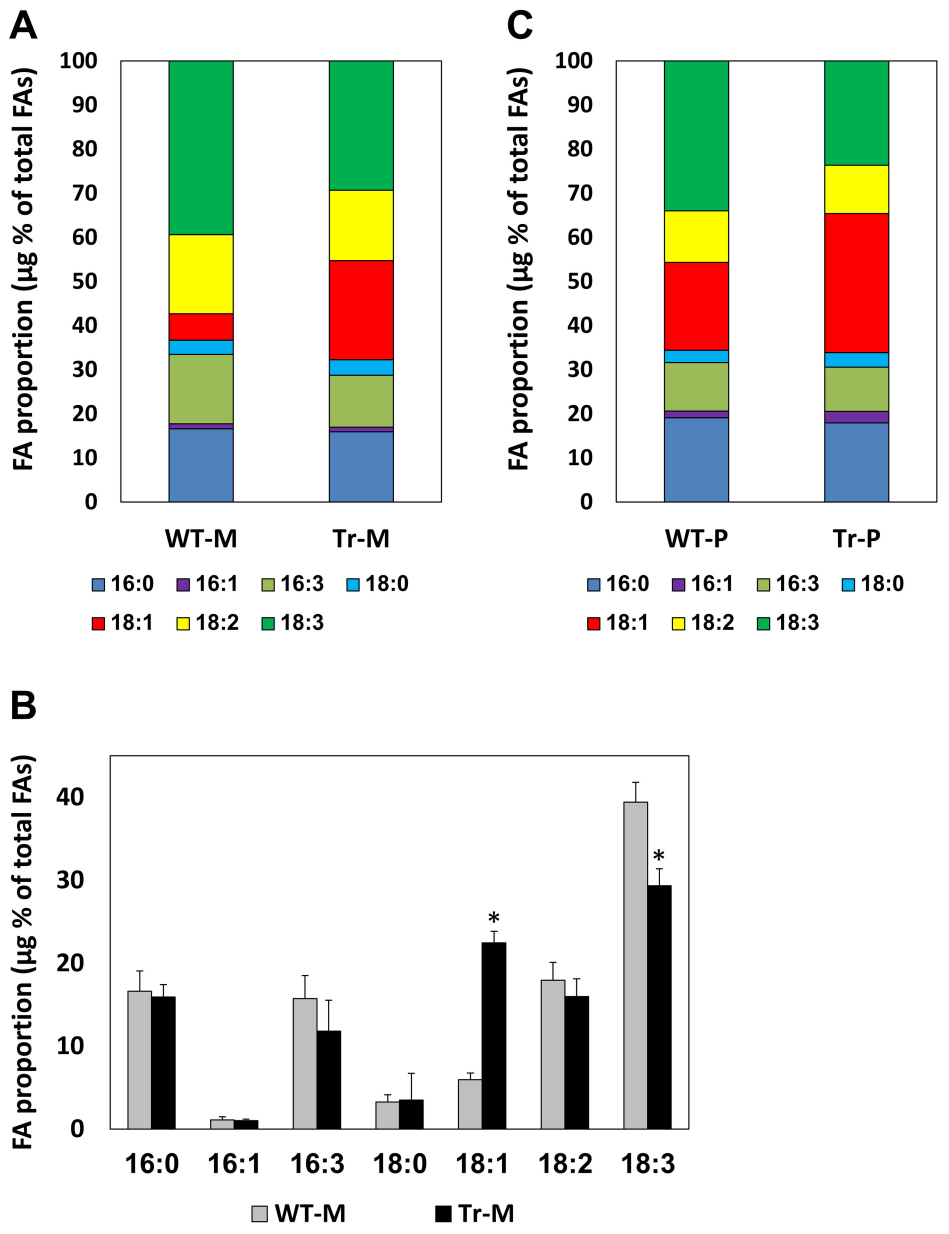
The FA profile of the algal lipids. The proportion of each FA is presented as the percentage of its content (in µg) from the total amount of lipid-derived FAs. **(A, B)** Algae grown five days in mixotrophic (M) conditions. The values shown are averages of four independent WT cultures (WT-M) and three independent transformed cultures (two of T6 and one of T89) (Tr-M). In **(B)**, the columns present the averages and standard errors of the data. An asterisk indicates a statistically significant difference (*p* < 0.05) between the WT and the transformed lines, as determined by two-sided Student’s *t*-test. **(C)** Algae grown 19 days in photoautotrophic (P) conditions. WT-P - untransformed cells, Tr-P - the T89 line.

The FA composition of the lipids affects the quality of biofuels. To determine the FA composition of the transformed algal lipids, the algae were grown in mixotrophic conditions, lipids were extracted and fatty acid methyl esters (FAMEs) acquired by transesterification were subjected to gas chromatography. The main FA observed in the algae were palmitic acid (16:0), palmitoleic acid (16:1), hexadecatrienoic acid (16:3), stearic acid (18:0), oleic acid (18:1), linoleic acid (18:2) and linolenic acid (18:3). Other FAs were present in minor amounts. **Figure 6** presents the abundance of each FA as a percentage of the total amount of lipid-derived FAs. The main change observed in the FA profile of the transformed algae was an about four fold increase in the proportion of oleic acid (18:1) - from 6% of the total FAs in WT algae to 23% in the transformed algae (**Figure 6A, B** and **Supplementary Figure S4**). This was compensated by a 25% reduction in the proportion of linolenic acid (18:3) in the transformed algae (**Figures 6A, B**). Both the increase in oleate levels and the reduction in linolenate levels are favorable for biodiesel production (Knothe, 2009) (see Introduction).

As delineated above, most of our experiments were carried out in mixotrophic conditions. However, we also tested the FA composition of lipids of algae grown in photoautotrophic conditions. Under these conditions, the proportion of oleic acid in WT algal lipids was 20% (**Figure 6C**) (see Discussion for a possible reason for the increased oleate proportion as compared to WT algae grown in mixotrophic conditions). Yet, the proportion of oleic acid in lipids of transformed algae grown under the same conditions was even higher - 32% (**Figure 6C**). This was accompanied by a reduction in the proportion of linolenic acid in the transformed algal lipids (**Figure 6C**). In fact, while linolenic acid was the most abundant FA in WT algal lipids, oleic acid became the most abundant FA in the lipids of transformed algae grown under photoautotrophic conditions (**Figure 6C**). This shift is favorable for biodiesel production.

## 4 Discussion

Based on the substrate specificity of AhDGAT3 to Oleoyl-CoA (Saha et al., 2006), we examined the ability of this enzyme to affect the neutral lipid content and FA profile of the oleaginous microalga *C. vulgaris*. Transformed *C. vulgaris* lines expressing AhDGAT3 showed up to a five fold increase in the content of neutral lipids compared to the WT algae. This significant increase in the neutral lipid content could result from the catalytic properties of AhDGAT3, and/or from our optimization of AhDGAT3 sequence and regulatory regions towards efficient expression.

Natural oleaginous microalgae produce high levels of neutral lipid (of which TAG are a major component) only under stress conditions such as nitrogen starvation (Hu et al., 2008). Our data show that AhDGAT3 overexpression can increase the neutral lipid content of *C. vulgaris*, under normal growth conditions, by about the same factor that could be obtained in WT algae under nitrogen starvation. Considering the reduction in culture biomass due to nitrogen starvation, the utilization of transformed algae and growth under nitrogen replete conditions is advantageous over the utilization of either the WT or transformed algae in growth processes that involves nitrogen starvation.

It was previously shown that mixotrophic cultivation of microalgae can elevate the biomass obtained compared to photoautotrophic conditions (Chen et al., 2018). The mixotrophic cultivation can overcome bottlenecks of photoautotrophy, including limitation in CO_2_ availability and light distribution, and is in most cases cheaper and simpler to maintain on a large scale (Perez-Garcia et al., 2011; Chen et al., 2018). The transformed *C. vulgaris* lines expressing AhDGAT3 showed increased neutral lipid levels as compared to the WT line under both the photoautotrophic and mixotrophic growth conditions. Yet, the growth rate of the transformed lines was higher in the mixotrophic compared to the photoautotrophic conditions, and the highest lipid levels were obtained in a much shorter period of time in the mixotrophic conditions (five days, as compared to more than two weeks in the photoautotrophic conditions).

The transformed algal lines expressing AhDGAT3 showed, under mixotrophic conditions, not only up to a five fold increase in the neutral lipid content but also an improved FA profile of the algal lipids. Compared to the WT line, the transformed algal lipids had an about four fold increase in the proportion of oleic acid (18:1), and about a 25% decrease in the proportion of linolenic acid (18:3). As explained in the Introduction, both changes are favorable for biodiesel production (Knothe, 2009).

In photoautotrophic conditions, the proportion of oleic acid in the WT algal lipids was 20%, which was close to that of transformed algae grown under mixotrophic conditions (23%) (**Figure 6**). Yet, it should be remembered that biomass accumulation is much more rapid under mixotrophic as compared to photoautotrophic conditions (Chen et al., 2018). One possible explanation for the increased proportion of oleic acid in lipids of WT algae grown under photoautotrophic as compared to mixotrophic medium is related to the long growth period (about 19 days) that was necessary to obtain maximal biomass and lipid content under the photoautotrophic conditions. It is possible that this long growth period resulted in a depletion of certain minerals, such as nitrogen, from the medium. It was previously shown that nitrogen starvation causes a significant increase in the proportion of oleic acid in lipids of WT *C. vulgaris* (Breuer et al., 2012).

Thus, overexpression of the peanut DGAT3 enzyme in the oleaginous microalga *C. vulgaris* resulted in a five fold increase in the neutral lipid content and a four fold increase in the proportion of oleic acid (18:1). Most previous attempts to overexpress DGAT enzymes in microalgae were carried out in the model non-oleaginous alga *C. reinhardtii*, and mainly involved DGAT2 enzymes. In most cases, DGAT2 overexpression shifted the algal FA profile towards polyunsaturated FAs, which are important for human nutrition but are less favorable for biodiesel production due to their high vulnerability to oxidation (Knothe, 2008; Liu, 2015). Expression of rapeseed (*Brassica napus*) DGAT2 in *C. reinhardtii* increased the algal neutral lipid content by two fold (Ahmad et al., 2015), with a significant increase in the proportion of linolenic acid (18:3). Expression of *C. reinhardtii* DGAT2 in this alga increased neutral lipid content by 50% under normal growth conditions (Deng et al., 2012), or by two fold when DGAT2 was expressed under a phosphate starvation inducible promoter and the algae were grown under phosphate starvation (Iwai et al., 2014). Expression of *Haematococcus lacustris* DGAT2D in *C. reinhardtii* resulted in a 40% increase in TAG content and a 23% increase in oleic acid (18:1) content, but this occurred under nitrogen deficient conditions (Cui et al., 2021). A few studies were also conducted in oleaginous algae. Expression in *Phaeodactylum tricornutum* of its own DGAT2 increased TAG levels by 35%, with a significant increase in the proportion of polyunsaturated FAs (Niu et al., 2013). Expression of rapeseed DGAT2 in *Chlorella sorokiniana*-I increased two fold the total lipid content of this microalga, with a 63% reduction in the content of oleic acid (18:1) (Nawkarkar et al., 2023).

A few studies also described the overexpression of DGAT1 enzymes in microalgae. Expression in *P. tricornutum* of its own DGAT1 enzyme resulted in a 2.3 fold increase in the TAG content, without affecting the FA profile (Zhang et al., 2021). Expression of *P. frutescens* DGAT1 in *C. reinhardtii* increased the TAG content by 32%, without affecting the FA profile (Zhou et al., 2025). Expression of *Auxenochlorella protothecoides* DGAT1 in *C. reinhardtii* increased the total lipid content by 11% and the oleic acid percentage by 60% (Liu et al., 2023). Expression of *A. thaliana* DGAT1 in the oleaginous microalgae *Nannochloropsis oceanica* increased the lipid content of this alga by 31% and elevated the percentage of palmitoleic acid (16:1) by 32% (Zhang et al., 2022). Overexpression in *N. oceanica* of its own DGAT1A enzyme resulted in a 50% increase in the TAG content under nitrogen replete conditions (Wei et al., 2017).

Higher plants DGAT3 enzymes had not been previously overexpressed in an oleaginous microalga. Certain algal proteins with a distant relation to higher plants DGAT3 enzymes were nominated DGAT3 in algae. These proteins included a DGAT clade exclusive to green algae, which showed some similarity to higher plants DGAT3s only in a carboxy-terminal region of a thioredoxin-like 2Fe-2S cluster-binding domain (Bagnato et al., 2017). These proteins shared a most recent ancestor with a group of uncharacterized cyanobacterial proteins. While higher plants DGAT3s are soluble cytosolic enzymes, most of the distantly related green algal DGAT3s are imported into the chloroplast, and participate in the plastidial pathway of TAG biosynthesis that is present in algae but not in higher plants (Bagnato et al., 2017). Expression of the *C. reinhardtii* DGAT3 in *E. coli* increased TAG accumulation in these bacteria (Bagnato et al., 2017). Diatoms are unicellular algae belonging to the class *Bacillariophyceae* (rather than the green algae group). Expression of the diatom *P. tricornutum* DGAT3 complemented a neutral lipid-deficient yeast mutant (Cui et al., 2013). Overexpression of the *P. tricornutum* DGAT1, DGAT2B, or DGAT3 enzymes in *P. tricornutum* increased TAG levels about two fold, but did not alter the FA composition of the TAG (Zhang et al., 2021). All these diatom DGATs included transmembrane domains and were localized at the chloroplast endoplasmic reticulum (cER). The cER (which is not present in green algae) is the outermost of the four plastidial membranes of *P. tricornutum*, which is fused with the ER and continuous with the outer nuclear envelope. Thus, the DGAT3 of the diatom *P. tricornutum* also differs from the cytosolic, non membranal DGAT3 of higher plants.

To conclude, this work demonstrates that expression of the peanut DGAT3 enzyme can significantly increase the neutral lipid content and improve the FA composition of the oleaginous microalga *C. vulgaris*. Oleaginous microalgae expressing AhDGAT3 can thus provide favorable microalgal feedstocks for biodiesel production. As mentioned (see Introduction), economic feasibility could be obtained by combining microalgal growth for biodiesel production with a second simultaneous utilization, such as industrial CO_2_ mitigation or wastewater remediation (Jayakumar et al., 2017; Chen et al., 2018; Fazal et al., 2018; Anahas et al., 2025).

## Data Availability

The original contributions presented in the study are included in the article/**Supplementary Material**. Further inquiries can be directed to the corresponding author.
