# Supplementary material for: Expression of the peanut diacylglycerol acyltransferase 3 increases the neutral lipid content and improves the fatty acid composition of Chlorella vulgaris
Source: Front Plant Sci. 2026 Mar 5;17:1750015. doi: 10.3389/fpls.2026.1750015 (PMC12999893; doi:10.3389/fpls.2026.1750015)
Supplement: Supplementary file 1 [file DataSheet1.pdf]

Supplementary material

NOS promoter

AGGCGGGAAACGACAATCTGATCATGAGCGGAGAATTAAGGGAGTCACGTTATGACC
CCCGCCGATGACGCGGGACAAGCCGTTTTACGTTTGGAACTGACAGAACCGCAACGA
TTGAAGGAGCCACTCAGCCGCGGGTTTCTGGAGTTTAATGAGCTAAGCACATACGTC
AGAAACCATTATTGCGCGTTCAAAAGTCGCCTAAGGTCACTATCAGCTAGCAAATAT
TTCTTGTCAAAAATGCTCCACTGACGTTCCATAAATTCCCCTCGGTATCCAATTAGA
GTCTCATATTCACTCTCAATCCAAATAATCTGCACCGGATCCCCTAGA

Sh ble

ATGGCCAAGTTGACCAGTGCCGTTCCGGTGCTCACCGCGCGCGACGTCGCCGGAGCG
GTCGAGTTCTGGACCGACCGGCTCGGGTTCTCCCGGGACTTCGTGGAGGACGACTTC
GCCGGTGTGGTCCGGGACGACGTGACCCTGTTCATCAGCGCGGTCCAGGACCAGGTG
GTGCCGGACAACACCCTGGCCTGGGTGTGGGTGCGCGGCCTGGACGAGCTGTACGCC
GAGTGGTCGGAGGTCGTGTCCACGAACTTCCGGGACGCCTCCGGGCCGGCCATGACC
GAGATCGGCGAGCAGCCGTGGGGGCGGGAGTTCGCCCTGCGCGACCCGGCCGGCAAC
TGCGTGCACTTCGTGGCCGAGGAGCAGGACTGA

Lo rbcS 3A ter

CCCAGCTTTCGTCCGTATCATCGGTTTCGACAACGTTCGTCAAGTTCAATGCATCAG
TTTCATTGCCCACACACCAGAATCCTACTAAGTTTGAGTATTATGGCATTGGAAAAG
CTGTTTTCTTCTATCATTTGTTCTGCTTGTAATTTACTGTGTTCTTTCAGTTTTTGT
TTTCGGACATCAAAATGCAAATGGATGGATAAGAGTTAATAAATGATATGGTCCTTT
TGTTCATTCTCAAATTATTATTATCTG

Zm Ubi1 promoter

TCTGCAGTGCAGCGTGACCCGGTCGTGCCCCTCTCCTAGAGATAATGAGCATTGCAT
GCCTAAGTTATAAAAAATTACCACATATTTTTTTTGTCACACTTGTTTGAAGTGCAG
TTTATCTATCTTTATACATATATTTAAACTTTACTCTACGAATAATATAATCTATAG
TACTACAATAATATCAGTGTTTTAGAGAATCATATAAATGAACAGTTAGACATGGTC
TAAAGGACAATTGAGTATTTTGACAACAGGACTCTACAGTTTTATCTTTTTAGTGTG
CATGTGTTCTCCTTTTTTTTTGCAAATAGCTTCACCTATATAATACTTCATCCATTT
TATTAGTACATCCATTTAGGGTTTAGGGTTAATGGTTTTTATAGACTAATTTTTTTA
GTACATCTATTTTATTCTATTTTAGCCTCTAAATTAAGAAAACTAAAACTCTATTTT
AGTTTTTTTATTTAATAATTTAGATATAAAATAGAATAAAATCTTGTTTCGAGTAGA
TAATGCCAGCCTGTTAAACGCCGTCGACGAGTCTAACGGACACCAACCAGCGAACCA
GCAGCGTCGCGTCGGGCCAAGCGAAGCAGACGGCACGGCATCTCTGTCGCTGCCTCT
GGACCCCTCTCGAGAGTTCCGCTCCACCGTTGGACTTGCTCCGCTGTCGGCATCCAG
AAATTGCGTGGCGGAGCGGCAGACGTGAGGCGGCACGGCAGGCGGCCTCCTCCTCCT
CTCACGGCACGGCAGCCTACGGGGGATTCCTTTCCCACCGCTCCTTCGCTTTCCCTT
CCTCGCCCGCCGTAATAAATAGACACCCCCTCCACACCCTCTTTCACC

At PhoA 5’UTR (bold capital letters) and *Cr rbcS* intron (small letters)

CTCGAGAAAAACAAAATAAAAAAACATCGCACAAGAAAATAAAAGATTTGTAGAATC
AACTACAGgtgagtcgacgagcaagcccggcggatcaggcagcgtgcttgcagattt
gacttgcaacgcccgcattgtgtcgacgaaggcttttggctcctctgtcgctgtctc
aagcagcatctaaccctgcgtcgccgtttccatttgcag**GAAACC**

Cr BiP1 SP

ATGATGAAGCTGTCCCTGAGCTTTCTGCTGCTGTTTCTGAGCTCCCTGGTGCAGGCA

Ah DGAT3

ATGGAGGTGAGCGGCGCAGTGCTGCGCAACGTGACATGCCCCTCCTTTAGCGTGCAC
GTGAGCTCCCGCCGCCGCGGGGGGACAGCTGCGTGACAGTGCCCGTTCGCATGCGC
AAGAAGGCAGTGGTGCGCTGCTGCTGCGGCTTTAGCGACAGCGGCCACGTGCAGTAC
TACGGCGACGAGAAGAAGAAGGAGAACGGCACAGCAATGCTGAGCACAAAGAAGAAG
CTGAAGATGCTGAAGAAGCGCGTGCTGTTTGACGATCTTCAGGGCAACCTGACATGG
GACGCAGCAATGGTGCTGATGAAGCAGCTGGAGCAGGTGCGCGCAGAGGAGAAGGAG
CTGAAGAAGAAGCGCAAGCAGGAGAAGAAGGAGGCAAAGCTGAAGGCAAGCAAGATG
AACACAAACCCCGACTGCGAGAGCAGCAGCAGCAGCAGCAGCAGCGAGAGCGAGAGC
GAGAGCAGCGAGAGCGAGTGCGACAACGAGGTGGTGGACATGAAGAAGAACATCAAG
GTGGGCGTGGCAGTGGCAGTGGCAGACTCCCCCCGCAAGGCAGAGACAATGATCCTG
TACACATCCCTGGTGGCACGCGACGTGAGCGCAAACCACCACCACCACAACGCAGTG
GAGCTGTTTAGCCGCAACAACGACATCAGCGTGGGCAGCATCAACGGCGGCCTGAAG
AACGAGAACACAGCAGTCATCACAACAGAGGCAATCCCCCAGAAGCGCATCGAGGTG
TGCATGGGCAACAAGTGCAAGAAGTCCGGCAGCATCGCACTGCTGCAGGAGTTTGAG
CGCGTGGTGGGCGCAGAGGGCGGCGCAGCAGCAGCAGTGGTGGGCTGCAAGTGCATG
GGCAAGTGCAAGAGCGCACCCAACGTGCGCATCCAGAACAGCACAGCAGACAAGATC
GCAGAGGGCTTTAACGACAGCGTGAAGGTGCCCGCAAACCCCCTGTGCATCGGCGTG
GCATGGCGCATGCTGAAGCCCCTGTGGCTGCGCTTTCTGGGCGAGAACCAGGAGAGC
ACAAACGAGAGCGCTCACGACGAGCTGTAA

*Cr rbcS*2 ter

GCTCCGTGTAAATGGAGGCGCTCGTTGATCTGAGCCTTGCCCCCTGACGAACGGCGG
TGGATGGAAGATACTGCTCTCAAGTGCTGAAGCGGTAGCTTAGCTCCCCGTTTCGTG
CTGATCAGTCTTTTTCAACACGTAAAAAGCGGAGGAGTTTTGCAATTTTGTTGGTTG
TAACGATCCTCCGTTGATTTTGGCCTCTTTCTCCATTGGGCGGGCTGGGCGTATTTG
AAGC

Supplementary Figure S1. The sequences synthesized by Biomatik for generating the chimeric genes. See the legend of Figure 1 for more details about the sequences.

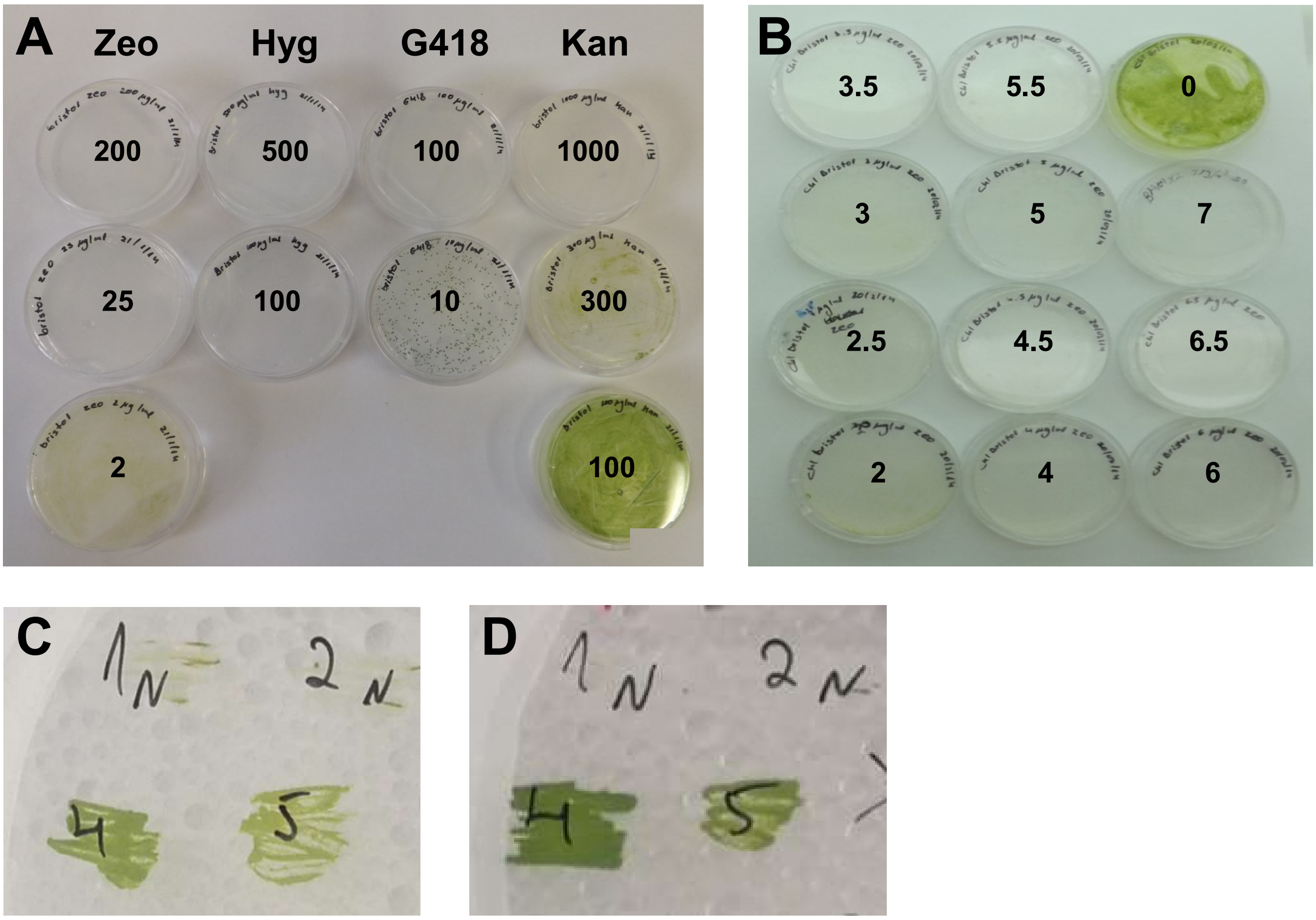

Supplementary Figure S2. *C. vulgaris* growth in agar plates including Bristol x2 medium and various concentrations of different antibiotics. (A) Growth in the presence of zeocin (Zeo), hygromycin (Hyg), G418, or kanamycin (Kan). (B) Growth in the presence of different concentrations of zeocin for identification of the minimal concentration that prevents the growth of WT algae. In (A) and (B), the numbers on each plate indicate the antibiotic concentration in μg ml^{-1}. (C, D) Growth of WT and transformed algae with 6 μg ml^{-1} zeocin (C) or 12 μg ml^{-1} zeocin (D). The test was performed few years after the transformed lines were obtained, to verify that the zeocin resistance was not lost. The numbers 1N and 2N indicate the position on the plates of the WT alga, 4 is the transformed T6 line, and 5 is the transformed T89 line.

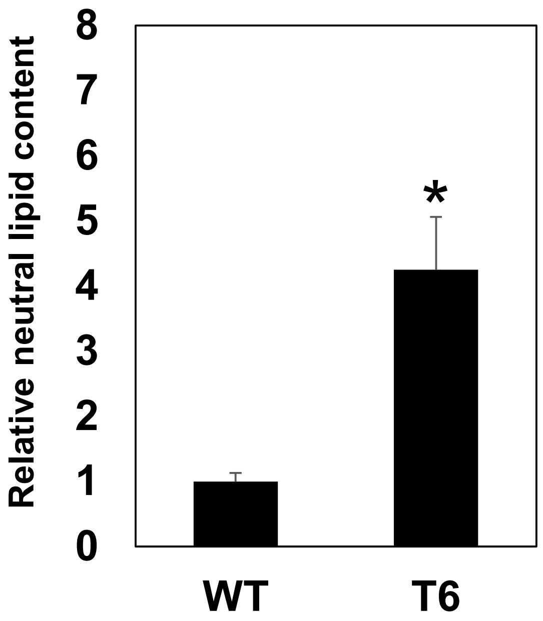

Supplementary Figure S3. Relative content of neutral lipids in the transformed algae. Algae were grown in photoautotrophic conditions for 14 days. The content of neutral lipids was determined as described in the Materials and Methods. The data were first normalized to the OD 750 nm of each culture (which is proportional to algal biomass) and then to the neutral lipid content of WT algae. The graph presents the averages and standard errors of three independent experiments; each experiment included three independent flasks containing 50 ml medium for each algal line. The asterisk indicates a statistically significant difference ($p<0.05$) between the WT and the transformed line, as determined by two-sided Student's *t*-test. WT - untransformed algae, T6 – a transformed algal line.

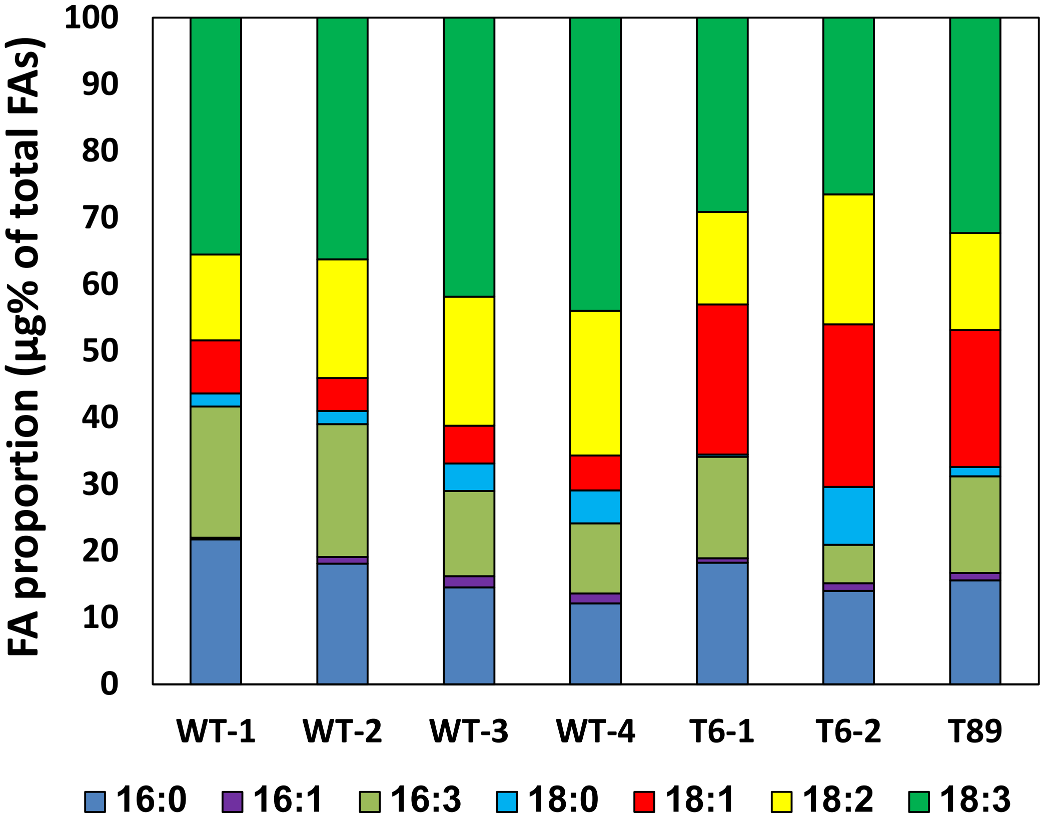

Supplementary Figure S4. The separate FA profiles that were averaged for the generation of Figure 6A. The proportion of each FA is presented as the percentage of its content (in µg) from the total amount of lipid-derived FAs. The algae were grown five days in mixotrophic conditions.
